# A Stochastic Phylogenetic Algorithm for Mitochondrial DNA Analysis

**DOI:** 10.3389/fgene.2019.00066

**Published:** 2019-03-08

**Authors:** M. Corona-Ruiz, Francisco Hernandez-Cabrera, José Roberto Cantú-González, O. González-Amezcua, Francisco Javier Almaguer

**Affiliations:** ^1^Facultad de Ciencias Físico-Matemáticas, Universidad Autónoma de Nuevo León, San Nicolás de los Garza, Mexico; ^2^Escuela de Sistemas PMRV, Unidad Acuña, Universidad Autónoma de Coahuila, Saltillo, Mexico

**Keywords:** DNA, random-walk, Hurst exponent, detrended fluctuation analysis, Shannon entropy, coefficient of disequilibrium

## Abstract

This paper presents an exploratory analysis of the mitochondrial DNA (mtDNA) of 32 species in the subphylum Vertebrata, divided in 7 taxonomic classes. Multiple stochastic parameters, such as the Hurst and detrended fluctuation analysis (DFA) exponents, Shannon entropy, and Chargaff ratio are computed for each DNA sequence. The biological interpretation of these parameters leads to defining a triplet of novel indices. These new functions incorporate the long-range correlations, the probability of occurrence of nucleic bases, and the ratio of pyrimidines-to-purines. Results suggest that relevant regions in mtDNA can be located using the proposed indices. Furthermore, early results from clustering algorithms indicate that the indices introduced might be useful in phylogenetic studies.

## 1. Introduction

Previous mathematical studies on DNA sequences have seen a variety of approaches and frequently involve a numerical representation of the nucleotide chains. For instance, distance matrices have been constructed using different metrics (Randi et al., 2003; Liao and Wang, 2004; Zhang and Tan, 2007; Kandiah and Shepelyansky, 2013). These matrices, in combination with clustering methods, are used to evaluate phylogenetic relationships among species (Yu and Huang, 2013).

Other studies involve the representation of DNA sequences as random-walks, known as *DNA-walks* (Peng et al., 1994). The main objectives of these studies focus on the long-range correlations among nucleotides; i.e., “how the frequency of each nucleotide of a pairing nucleotide couple changes locally” (Namazi and Kiminezhadmalaie, 2015). These DNA-walk studies find differences in the long-range correlation between coding and non-coding DNA sequences (Peng et al., 1994).

Recently, DNA-walk analysis has been used in combination with the fractal dimension and Hurst exponent to identify mosaic structures in DNA that allow distinguishing between healthy and cancerous cells (Namazi and Kiminezhadmalaie, 2015).

Additionally, alternative statistical tools frequently used in DNA sequence analysis include Shannon entropy, which is a measure of the amount of “information" stored within a system (López-Ruiz et al., 1995). In a biological sense, Shannon entropy evaluates the probability of independent occurrences of each nucleic base in a DNA sequence. In recent studies, fluctuations in local Shannon entropy in DNA sequences have been analyzed to identify regions of repeating patterns of one or more nucleotides, known as *tandem repeats* (Thanos et al., 2018). The capability of Shannon entropy to highlight important segments in DNA sequences has led to the supported notion that entropy studies might be used for biological classifications of species (Melnik and Usatenko, 2014).

Similarly, the concept of complexity has played a central role in various DNA sequence analyses. For instance, López-Mancini-Calbet (LMC) complexity, employed in this paper, has led to the development of an effective gene-predicting technique (López-Ruiz et al., 1995; Monge and Crespo, 2015). In a recent study, the symbolic complexity of DNA sequences is used to identify segments resulting from random duplication, as well as changes in the speed of accumulation of point mutations (Salgado-Garcia and Ugalde, 2016).

Our objective is to examine the parameters previously mentioned to determine a small number of coefficients with biological relevance that may be used to determine rates of change in nucleotide bases, establish comparisons between regions, and better understand the relation among species in a phylogenetic sense.

This paper is structured as follows: section 2 introduces the concepts and methodology; section 3 presents the results obtained and the variables introduced; and section 4 is devoted to a discussion of the results, comments on the methodology in general, and final remarks. Tables and figures are incorporated in sections 2 and 3, respectively. The Supplementary Material includes a table with the identification codes for the data.

## 2. Methodology

GenBank^®^ is the *National Institutes of Health*'s genetic sequence database made possible by the collaboration of several organizations. All datasets used within this work were obtained through GenBank because of its availability of access, encouragement of use, and the advantage that the information stays up-to-date.

A total of 32 complete mtDNA sequences of different species in the subphylum Vertebrata were selected. The lengths vary from 16, 207 to 18, 254 base pairs (bp). The choice of this type of DNA presents multiple advantages: it is relatively small in size (in contrast, human chromosomal DNA contains hundreds of millions bp); the sequences contain conserved regions, can be compared in blocks among different species, and contain a small percentage of non-coding regions; and the interpretation of the mutations in mtDNA as an estimator of evolutionary change (Barton and Jones, 1983). For these reasons, the exploratory nature of this study does not require additional information on the species themselves. Thus, the selection criteria focused on 32 different members from 7 groups intuitively related in taxonomic classes. The 32 NCBI codes from the data files have been attached in the Table S1.

A pre-processing of the data files consists of a realignment of the sequences to set the control region of the heavy chain (H-chain) in the direction of transcription as the new ending point. This realignment is done once. The *displacement loop*, or D-loop, is within the control region and the most varying region in mtDNA, with substantial differences observed even among individuals of the same species (Yamamoto, 2001). See Figure S1 (Supplementary Material). Additionally, the header information was removed, which contains the identification key and the name of the organism. The downloaded files (in *.fasta* format) were processed using the programming language R version 3.4.4 (2018-03-15). The packages used are *stringr* and *fractal*.

### 2.1. DNA-Walk

**Figure 1:**
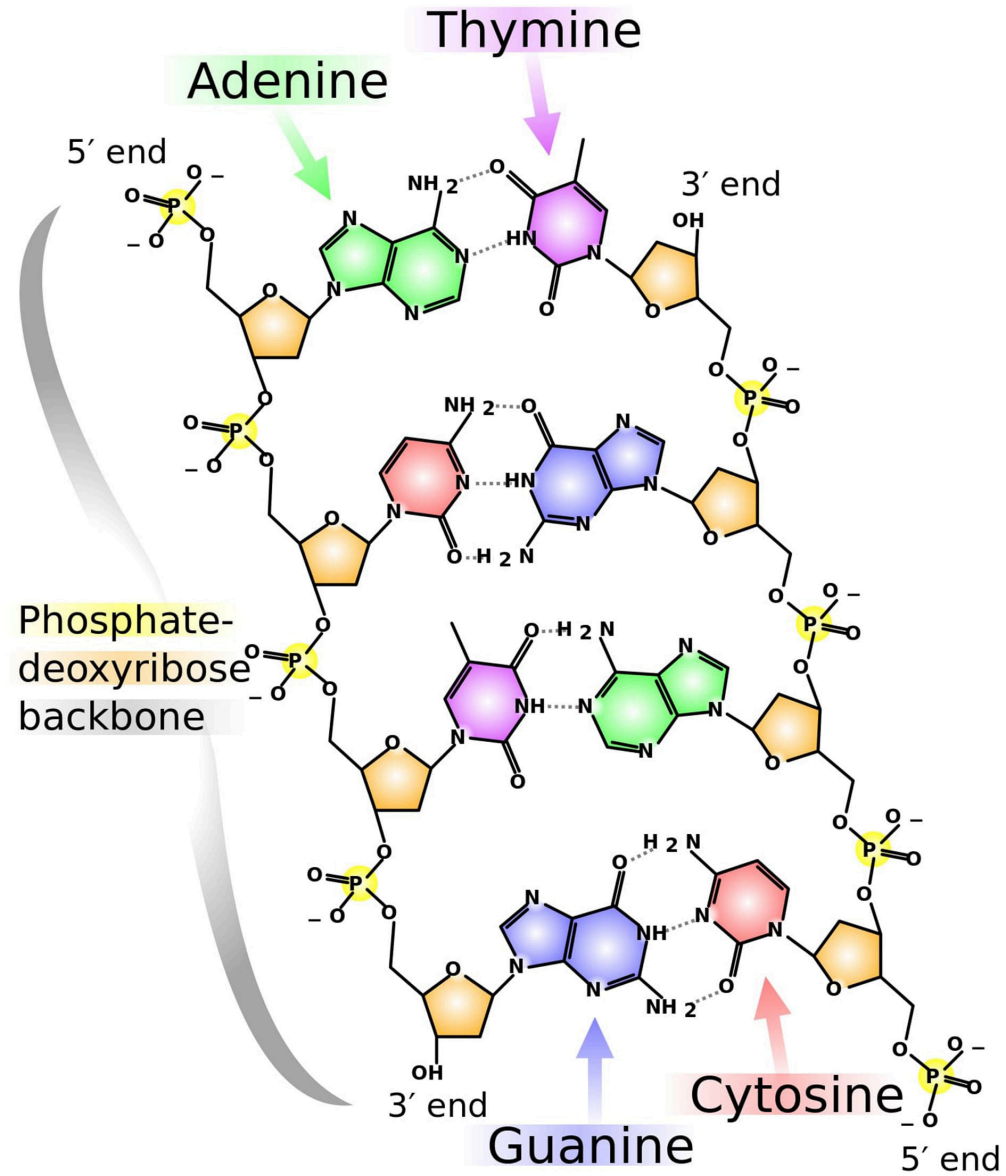
Chemical structure of DNA. Adenine, guanine, cytosine, and thymine are shown in colors green, blue, red, and purple, respectively. Notice the double-ring structure of the purines (*A, G*) and the single-ring structure of the pyrimidines (*C, T*). Similarly, the type of bond is readily observable: double- and triple-Hydrogen bonds for *A, T* and *G, C*, respectively. This illustration, by Madeleine Price Ball, has a Creative Commons Zero (CC0, i.e., “No Rights Reserved") license and has been published in previous articles (Wikimedia Commons Contributors, 2018).

DNA consists of sequences of nitrogenous bases: adenine (*A*), guanine (*G*), thymine (*T*), and cytosine (*C*). The length and distribution of the bases fluctuate from species to species. Several mappings have been introduced based on properties intrinsic to DNA. Moreover, adenine and guanine have a two-ring structure and belong to the *purine* group, while cytosine and thymine have a one-ring structure and belong to the *pyrimidine* group. Furthermore, adenine bonds with thymine through a double hydrogen bond, which is called a *weak bond*, while guanine and cytosine bond through a triple hydrogen bond, which is called a *strong bond*. Figure 1 illustrates these descriptions. In summary, we have:

- *Purine (R)*: {*A, G*} / *Pyrimidine (Y)*: {*C, T*}
- *Strong Hydrogen bond (S)*: {*G, C*} / *Weak Hydrogen bond (W)*: {*A, T*}
- *Keto (K)*: {*G, T*} / *Amino (M)*: {*A, C*}

Considering the properties described previously, it is possible to read a DNA sequence and assign either a +1 or −1 depending on whether the respective nucleotide is a purine or pyrimidine (*RY* rule). This can be interpreted as random steps *x*_*i*_ of a one-dimensional walk. Then, the final position after *n* steps is given by

(1) $$\begin{array}{r} X_{n}=x_{0}+\overset{n}{\underset{i=1}{\sum }}x_{i} \end{array}$$

where *x*_0_ = 0 by definition.

Let *S* = {*s*_1_*s*_2_…*s*_*M*_} be a nucleotide sequence of length *M*, where *s*_*k*_ ∈ {*A, C, G, T*} for *k* ∈ {1, 2, …, *M*}. Hence, a one-dimensional DNA-walk can be defined through the following rules:

- *RY rule:*
  (2) $$x_{k}=\{\begin{array}{ll} 1 & \text{ if }s_{k}\in R=\{A,G\}\\ -1 & \text{ if }s_{k}\in Y=\{C,T\} \end{array}$$
- *SW rule:*
  (3) $$x_{k}=\{\begin{array}{ll} 1 & \text{ if }s_{k}\in S=\{C,G\}\\ -1 & \text{ if }s_{k}\in W=\{A,T\} \end{array}$$
- *KM rule:*
  (4) $$x_{k}=\{\begin{array}{ll} 1 & \text{ if }s_{k}\in M=\{A,C\}\\ -1 & \text{ if }s_{k}\in K=\{G,T\} \end{array}$$

where *s*_*k*_ is the *k*−th nucleotide and *x*_*k*_ is the value of the *k*−th assigned step in a DNA sequence. The path of the DNA-walk after *n* steps is then defined as the partial sums $X_{n}=x_{0}+\overset{n}{\underset{k=1}{\sum }}x_{k}$, where *n* ∈ {1, 2, …, *M*} and *x*_0_ = 0.

In the context of DNA-walks, Equation (2) evaluates the tendency of changes between purines and pyrimidines. Transversions (substitutions of purines for pyrimidines, or vice versa) are less likely to happen and have been used to evaluate molecular evolution (Stoltzfus and Norris, 2016). Thus, using this rule within corresponding blocks of nucleotides in different species, it is possible to observe changes in the DNA-walk that could be interpreted as an evolutionary variation. Similarly, Equation (4) is associated with the rate of recombination between transversions and transitions (purine-purine or pyrimidine-pyrimidine substitutions).

Moreover, Equation (3) refers to the difference in abundance of the *GC* bond with respect to the *AT* bond. A higher *GC* content suggests a significantly higher temperature for DNA denaturing (melting temperature *T*_*m*_). Previous studies have shown that *GC* content is associated to an age-related natural selection and environmental factors (Min and Hickey, 2008). Finally, it is assumed that each DNA-walk is an ergodic stochastic process. Specifically, the conceived notion adopted is that each DNA sequence may be used to represent the ensemble of DNA sequences of individuals within the same species.

In summary, the three assignment rules provide insight into the evolutionary aspects of the organisms considered.

### 2.2. Hurst Exponent and DFA Exponent

Additional information of the long-range correlations of DNA-walks can be obtained via stochastic methods such as *rescaled-range analysis* and *detrended fluctuation analysis*. With these methods, it is possible to obtain the Hurst exponent, which represents a quantitative measure of the fractal nature of DNA sequences.

The Hurst exponent, here denoted by α, satisfies 0 < α < 1. In comparisons of mtDNA sequences, each Hurst exponent can be interpreted as a measure of the tendency of changes between nucleotides according to the rules mentioned in the previous section. The calculations used to obtain the Hurst exponent have been reported in previous studies (Peng et al., 1994; Buldyrev et al., 1995).

The Hurst exponent is directly related to the fractal dimension α′ by the relation:

(5) $$\begin{array}{r} \alpha^{\prime }=2-\alpha . \end{array}$$

The fractal dimension evaluates changes in detail of the pattern of a DNA-walk with respect to the scale used for measurement.

An alternative method to calculate the Hurst exponent of a DNA-walk is DFA. In contrast to the rescaled-range analysis, DFA analyzes the random fluctuations of the DNA-walk without trend in the data (Peng et al., 1994; Buldyrev et al., 1995). The DFA exponent is computed using the following algorithm:

- Given a numerical sequence *X* = {*X*_1_, *X*_2_, …, *X*_*M*_}, calculate the cumulative sum
  (6) $$\begin{array}{r} y_{k}=\overset{k}{\underset{i=1}{\sum }}\left(X_{i}-\bar{X}\right) \end{array}$$
  where *k* = 1, 2, …, *M* and $\bar{X}$ is the mean value of *X*.
- Divide *y*_*k*_ into *M*/*L* subintervals of length *L*. For each window, calculate the polynomial linear fit (the local trend) *y*_*k, L*_ via least-squares minimization.
- Calculate the fluctuation, which is an average of the squares of the detrended sequence given by
  (7) $$F^{2}(L)=\frac{1}{M}\sum_{k=1}^{M}|y_{k}-y_{k,L}{|}^{2}.$$
- The slope β of the linear regression analysis in the scale log*F*(*L*)/log*L* is an estimator of the Hurst exponent.

This method tests for self-similarity at different window sizes *L*. No correlation (or short-range correlations) gives stochastic properties such as those of a random-walk, so β = 0.5; in contrast, long-range correlations give a value of β ≠ 0.5. Specifically, correlation yields β > 0.5, while anti-correlation gives β < 0.5.

This paper adopts a minimum block size of 4 nucleotides, while the maximum is $B=\frac{M}{2}$, corresponding to half the length of the sequence in question. Should *M* be odd, *B* is rounded down.

### 2.3. Chargaff Ratio

In a remarkable discovery, Erwin Chargaff determined that there is a balance held in DNA by the nucleobases (Chargaff, 1950), known as *Chargaff's Rule*. These state: (1) that globally (i.e., considering both strands of DNA) adenine is equal to thymine in quantity, and (2) that guanine is equal to cytosine in quantity. This result was the basis for the Watson-Crick model, which determined that adenine binds with thymine and that guanine binds with cytosine (Watson and Crick, 1953).

On this basis, and in the context of this work, the *Chargaff ratio* is defined as the ratio of pyrimidines to purines:

(8) $$\xi =\frac{N_{C}+N_{T}}{N_{A}+N_{G}}$$

where *N*_*C*_, *N*_*T*_, *N*_*A*_, *N*_*G*_ represent the amount of cytosine, thymine, adenine, and guanine, respectively, within one strand of DNA. Note that this value is always positive. If 0 ≤ ξ < 1, there are more purines than pyrimidines (i.e., *N*_*C*_ + *N*_*T*_ < *N*_*A*_ + *N*_*G*_); similarly, ξ > 1 reflects an excess of pyrimidines over purines. A Chargaff ratio with value 1 results from an equal number of either type of nucleotide bases.

### 2.4. Shannon Entropy

In his seminal paper, Claude Shannon introduced the concept of *information entropy*. It measures the “amount" of information or uncertainty of a system (Shannon and Weaver, 1998). Let Ω = {ω_1_, ω_2_, …, ω_*N*_} be a set of events where each ω_*i*_ has probability of occurrence *p*_*i*_ ∈ [0, 1], for *i* = 1, 2, …, *N*. Thus, the Shannon entropy of the system is defined as

(9) $$\begin{array}{r} H=-K\overset{N}{\underset{i=1}{\sum }}p_{i}\log_{2}\left(p_{i}\right), \end{array}$$

where *K* is a positive constant chosen appropriately according to the units desired for measurement (thus, for this work, *K* = 1). For the case when *p*_*i*_ = 0, *p*_*i*_ log_2_(*p*_*i*_) = 0 in the limit definition. Also, note that the logarithm is in base 2; this is because information in a computer is encoded in *binary digits*, or *bits*, which are the basic units of measurement of information.

For *N* = 2, events ω_1_ and ω_2_ have probability *p* and 1−*p*, respectively, see Figure S2 (Supplementary Material). Thus, it can be seen that a maximum is attained at $p=1-p=\frac{1}{2}$. This result can be extended to the general case with *N* events. The proof requires *Jensen's inequality* for a concave function (in this case, the *logarithmic* function), and is given below. Using some algebra to rewrite Equation (9) with *K* = 1 yields

$$H=\log_{2}\left(\overset{N}{\underset{i=1}{\prod }}{\left(\frac{1}{p_{i}}\right)}^{p_{i}}\right)$$

By the *weighted arithmetic-mean and geometric-mean* inequality, this implies that

$$2^{H}=\overset{N}{\underset{i=1}{\prod }}{\left(\frac{1}{p_{i}}\right)}^{p_{i}}\leq \overset{N}{\underset{i=1}{\sum }}p_{i}\left(\frac{1}{p_{i}}\right)=N$$

where equality (the maximum) is satisfied when *p*_1_ = *p*_2_ = ⋯ = *p*_*N*_. That is, when

(10) $$\begin{array}{r} H=\log_{2}\left(N\right). \end{array}$$

To evaluate Shannon entropy in the context of DNA sequence analysis, it seems rather reasonable to define the set of possible events as Ω = {*A, G, C, T*}. However, it is expected that the probability of occurrence of each nucleotide in a DNA sequence will likely be different for different species; thus, these associated probabilities will be calculated empirically for each DNA sequence in a straightforward fashion. That is, by counting the amount of each nucleotide within the sequence and taking the corresponding proportion by dividing by the total amount of nucleotides *M*. Thus, the probabilities will be given by

(11) $$\begin{array}{r} p_{A}=\frac{N_{A}}{M},\;p_{C}=\frac{N_{C}}{M},\;p_{G}=\frac{N_{G}}{M},\;p_{T}=\frac{N_{T}}{M}, \end{array}$$

where *N*_*A*_, *N*_*C*_, *N*_*G*_, *N*_*T*_ are the amount of *adenine, cytosine, guanine*, and *thymine*, respectively.

In the context of DNA sequence analysis, maximum entropy is attained whenever the nucleic bases within a DNA sequence are found with equiprobability. It may thus be interpreted that such a sequence is the result of a random combination of these events. Any departure from the maximum value of the Shannon entropy due to an underlying structure might contribute to determining any tendencies present in a sequence, see Figure S3 (Supplementary Material).

In a more general sense, the entropy fluctuations could be analyzed by means of the Local Shannon entropy. By studying the local fluctuations of entropy at a given scale, and across scales, an “entropic microscope" could highlight areas with a high degree of variation or, equally interesting, low degree of variation, as seen in previous studies (Melnik and Usatenko, 2014; Thanos et al., 2018).

### 2.5. Coefficient of Disequilibrium

Additional information of DNA sequences can be derived from the deviations from equiprobability of occurrence of each nucleotide. This measure is known as *disequilibrium* (López-Ruiz et al., 1995). The events in the set Ω have probability *p*_*i*_ for *i* = 1, 2, 3, 4. The coefficient of disequilibrium, D, is defined as:

(12) $$\begin{array}{r} D=\overset{N=4}{\underset{i=1}{\sum }}{\left(p_{i}-\frac{1}{4}\right)}^{2}. \end{array}$$

This sum of squared distances can be seen as a type of variance. Note that D=0 in the case of equilibrium. Any deviation from this would result in D>0. The maximum disequilibrium value, $D_{\text{max}}=\frac{3}{4}$ can be obtained using multivariate calculus.

The coefficient of disequilibrium may represent a measure of relatedness between a DNA sequence and one resulting from a random process if each (independent) event has a probability *p*_*i*_ of occurrence. That is, larger deviations from an equiprobable space yield higher coefficients of disequilibrium. It can be observed that this behavior counters that of the Shannon entropy in an intuitive manner.

### 2.6. Coefficient of Complexity

The coefficient of complexity C is then given by the product of the Shannon entropy (9) and the coefficient of disequilibrium (12), as in (13). It can be seen from (12) that D resembles the definition of variance; thus, the coefficient of complexity can be interpreted as a measure of dispersion within the information stored in a system (López-Ruiz et al., 1995).

(13) $$\begin{array}{r} C=\mathrm{HD}=\left(-\overset{N}{\underset{i=1}{\sum }}p_{i}\log_{2}\left(p_{i}\right)\right)\left(\overset{N}{\underset{i=1}{\sum }}{\left(p_{i}-\frac{1}{N}\right)}^{2}\right). \end{array}$$

The coefficient of complexity may thus be regarded as the Shannon entropy weighted by the coefficient of disequilibrium, which can be interpreted as the tendency of a random sequence.

## 3. Results

**Figure 2:**
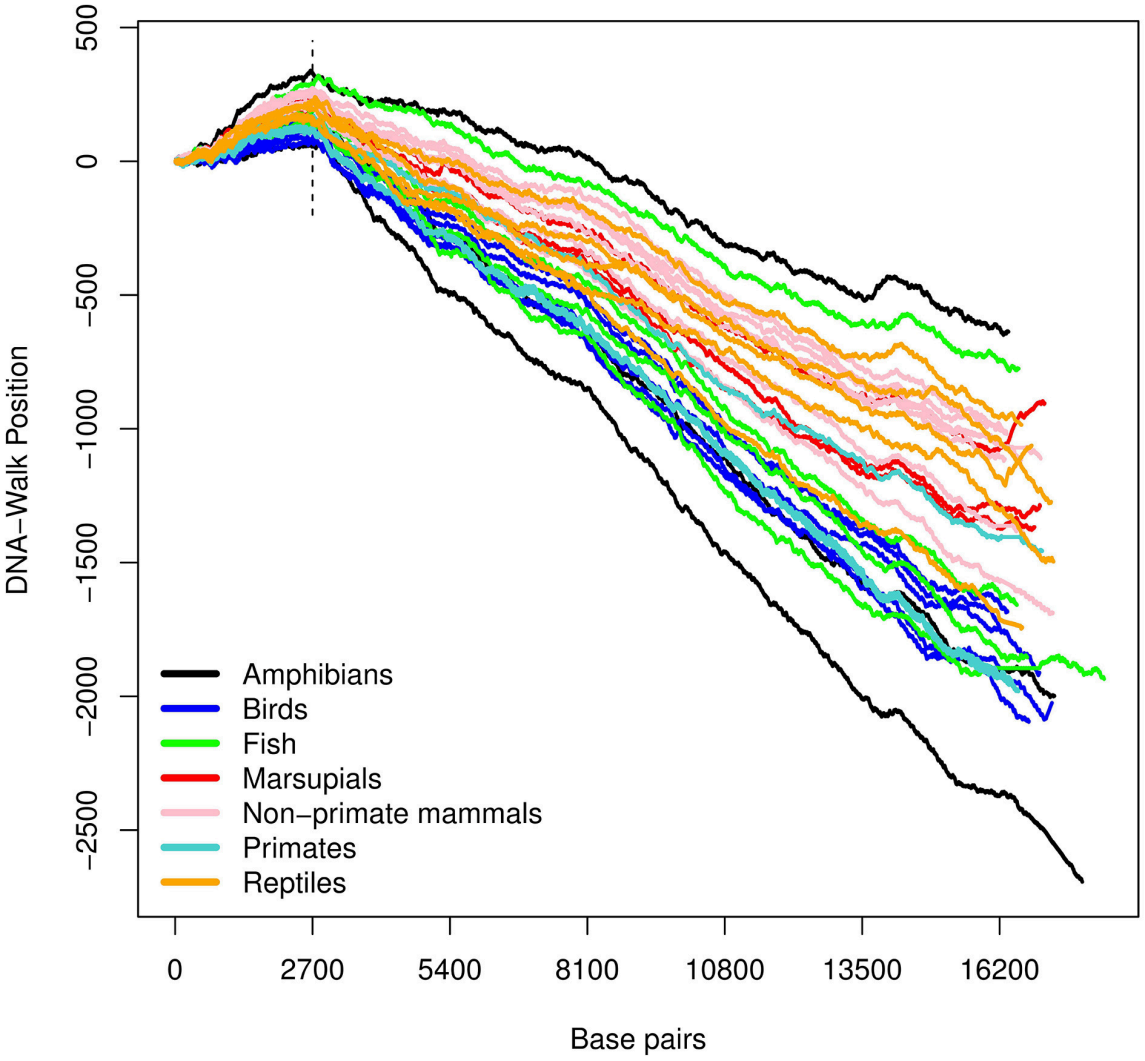
DNA-walk illustration for various species using the *purine-pyrimidine* rule. Observe the vicinity of nucleotide 2, 700 and the change in tendency from a purine-rich region (positive slope) to a predominance of pyrimidines for the remaining DNA-walk (negative slope).

**Figure 3:**
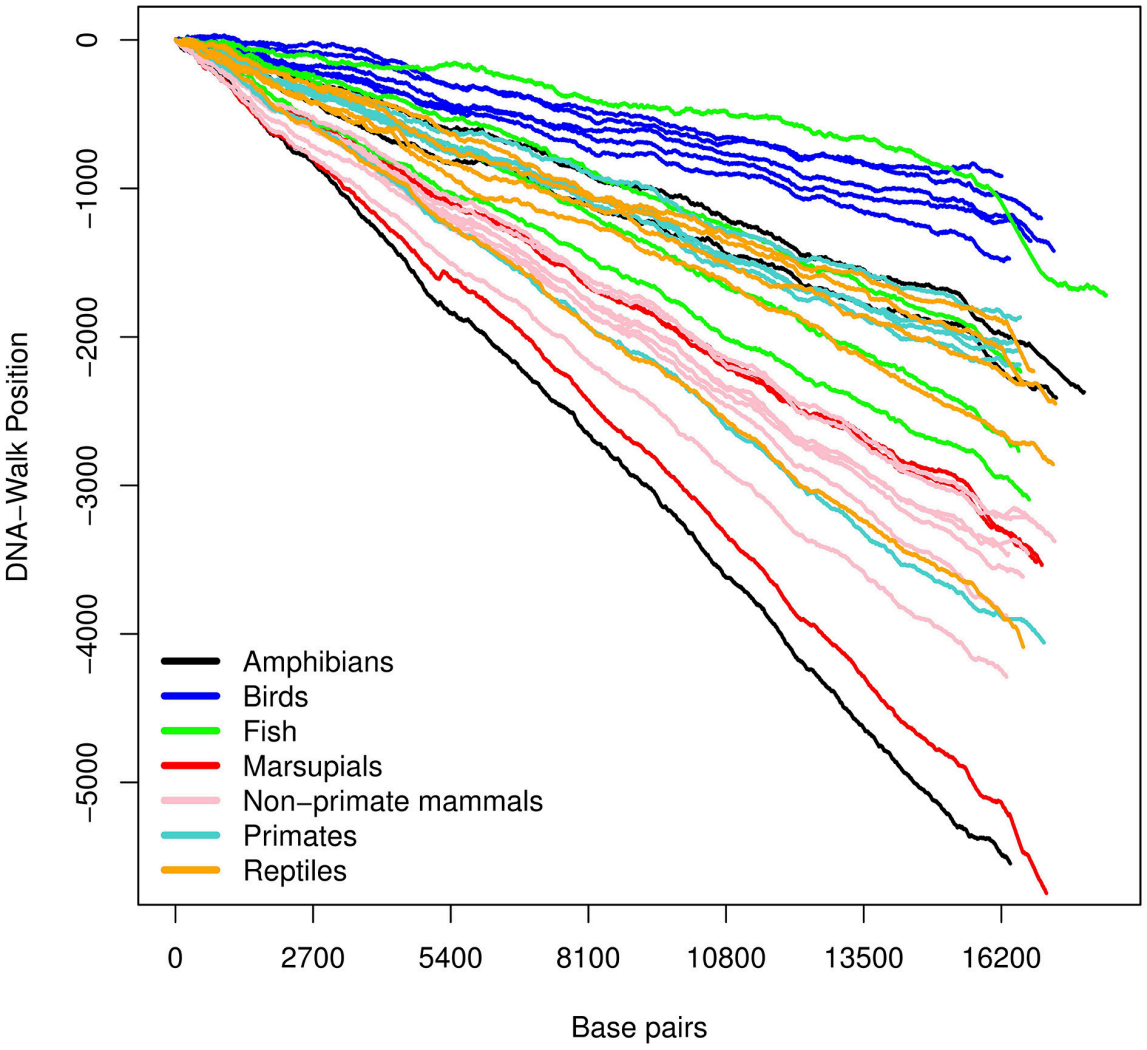
DNA-walk illustration for various species using the *strong- and weak-bond* rule. Observe the immediate (and consistent) tendency. This indicates that mtDNA is rich in adenine and thymine, whose type of bond is weaker than that of cytosine and guanine.

**Figure 4:**
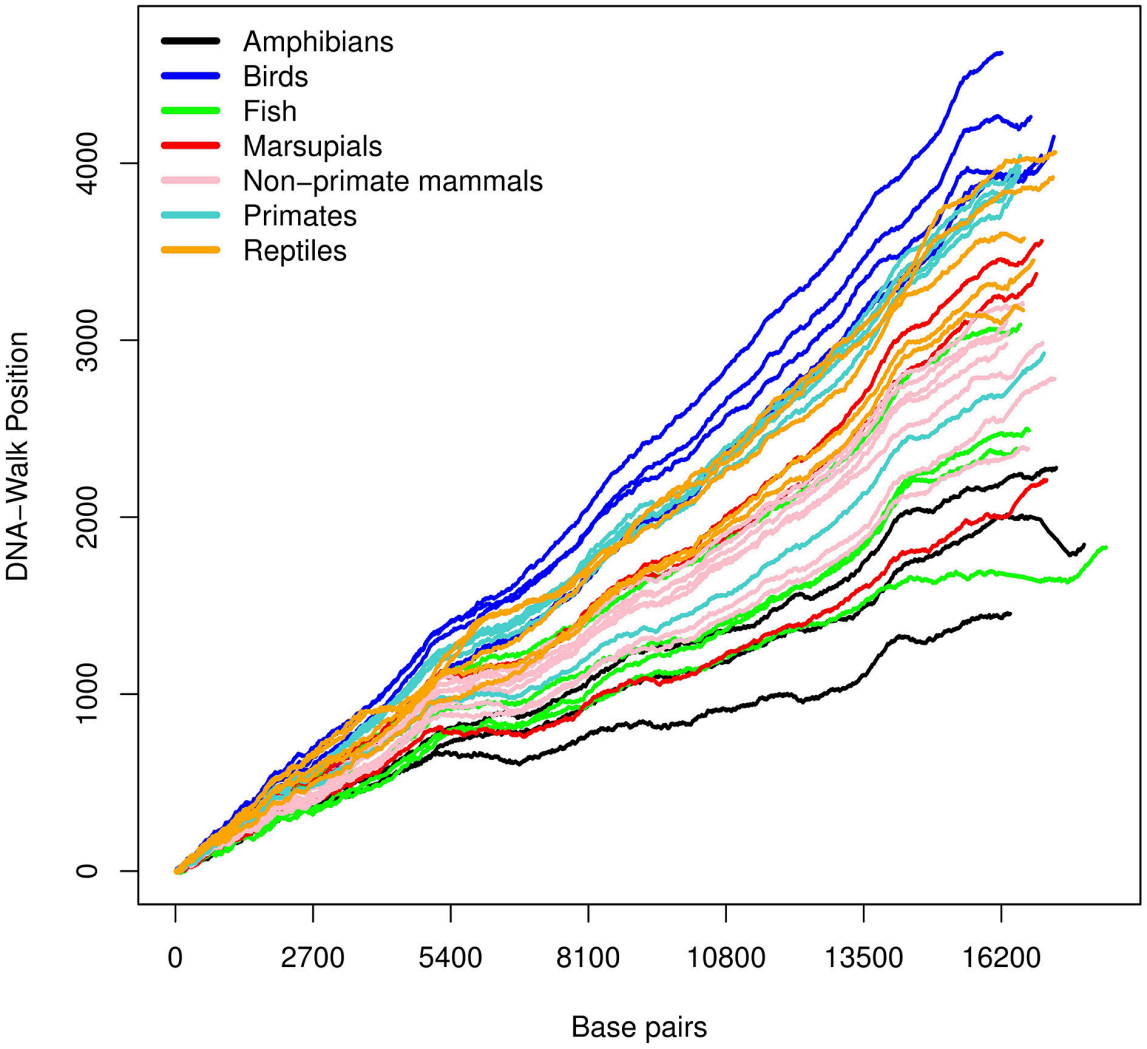
DNA-walk illustration for various species using the *keto and amino* rule. The figure shows a higher amount of adenine and cytosine.

The three DNA-walks for the 7 groups are depicted in Figures 2–4. Results for the Chargaff ratio ξ and Shannon entropy H are shown in Table 1, while Tables 2, 3 contain the Hurst and DFA exponents for each type of random-walk and for each sequence.

**Table 1:** Results of the Chargaff ratio and Shannon entropy for all groups.

**Scientific name (common name)**	**ξ**	**H**
*Ambystoma tigrinum tigrinum* (Eastern tiger salamander)	1.081	1.9059
*Bufo gargarizans* (Chusan Island toad)	1.2617	1.9598
*Rana plancyi* (Eastern golden frog)	1.3562	1.9591
*Ara ararauna* (Blue-and-yellow macaw)	1.2537	1.9421
*Archilochus colubris* (Ruby-throated hummingbird)	1.2296	1.9409
*Columba livia* (Rock pigeon)	1.2664	1.9381
*Gallus gallus* (Red junglefowl)	1.2851	1.9316
*Ninox strenua* (Powerful owl)	1.2421	1.926
*Carcharodon carcharias* (Great white shark)	1.249	1.9444
*Cyprinus carpio* (Common carp)	1.0981	1.9577
*Dicentrarchus labrax* (European seabass)	1.2372	1.9765
*Poecilia reticulata* (Guppy)	1.2228	1.9529
*Didelphis virginiana* (Virginia Opossum)	1.1117	1.8969
*Macropus giganteus* (Eastern gray kangaroo)	1.1762	1.9275
*Vombatus ursinus* (Common wombat)	1.164	1.9254
*Bos taurus* (Cattle)	1.1332	1.9339
*Canis lupus familiaris* (Dog)	1.1848	1.9441
*Capra aegagrus* (Wild goat)	1.1441	1.9292
*Felis catus* (Domestic cat)	1.1398	1.9429
*Mus musculus musculus* (House mouse)	1.1316	1.9154
*Oryctolagus cuniculus* (Common rabbit)	1.2169	1.9403
*Rattus rattus* (House rat)	1.1465	1.9219
*Gorilla gorilla gorilla* (Western lowland gorilla)	1.2706	1.9322
*Homo sapiens* (Human)	1.2716	1.9305
*Lemur catta* (Ring-tailed lemur)	1.1869	1.9246
*Pan paniscus* (Bonobo)	1.2711	1.9272
*Pan troglodytes* (Common chimpanzee)	1.2717	1.9293
*Alligator mississippiensis* (American alligator)	1.2338	1.9383
*Chelydra serpentina* (Common snapping turtle)	1.1259	1.9205
*Crocodylus niloticus* (Nile crocodile)	1.1347	1.9504
*Crotalus horridus* (Timber rattlesnake)	1.1898	1.9337
*Naja naja* (Indian cobra)	1.1597	1.9324

**Table 2:** Results of the Hurst exponent for all groups and each of the three random-walk rules.

**Scientific name (common name)**	**α_*RY*_**	**α_*SW*_**	**α_*KM*_**
*Ambystoma tigrinum tigrinum* (Eastern tiger salamander)	0.91798	0.91328	0.90701
*Bufo gargarizans* (Chusan Island toad)	0.91688	0.91187	0.91191
*Rana plancyi* (Eastern golden frog)	0.91695	0.91259	0.91228
*Ara ararauna* (Blue-and-yellow macaw)	0.91657	0.91337	0.9133
*Archilochus colubris* (Ruby-throated hummingbird)	0.91621	0.91298	0.91332
*Columba livia* (Rock pigeon)	0.91696	0.91336	0.91383
*Gallus gallus* (Red junglefowl)	0.91564	0.91109	0.91368
*Ninox strenua* (Powerful owl)	0.91569	0.91662	0.91341
*Carcharodon carcharias* (Great white shark)	0.91506	0.91385	0.91056
*Cyprinus carpio* (Common carp)	0.91759	0.91463	0.91045
*Dicentrarchus labrax* (European seabass)	0.91881	0.90116	0.91412
*Poecilia reticulata* (Guppy)	0.91631	0.91447	0.90864
*Didelphis virginiana* (Virginia Opossum)	0.91844	0.91408	0.90997
*Macropus giganteus* (Eastern gray kangaroo)	0.91811	0.91388	0.91113
*Vombatus ursinus* (Common wombat)	0.9179	0.91391	0.91207
*Bos taurus* (Cattle)	0.91704	0.9137	0.91125
*Canis lupus familiaris* (Dog)	0.91666	0.91426	0.91009
*Capra aegagrus* (Wild goat)	0.91783	0.9136	0.91174
*Felis catus* (Domestic cat)	0.91755	0.91438	0.91172
*Mus musculus musculus* (House mouse)	0.91641	0.91368	0.91138
*Oryctolagus cuniculus* (Common rabbit)	0.91665	0.91411	0.91117
*Rattus rattus* (House rat)	0.91655	0.91301	0.9119
*Gorilla gorilla gorilla* (Western lowland gorilla)	0.91509	0.91436	0.91224
*Homo sapiens* (Human)	0.91549	0.91484	0.91255
*Lemur catta* (Ring-tailed lemur)	0.91821	0.91424	0.91033
*Pan paniscus* (Bonobo)	0.91545	0.91465	0.91235
*Pan troglodytes* (Common chimpanzee)	0.91548	0.9146	0.91225
*Alligator mississippiensis* (American alligator)	0.91704	0.91213	0.91343
*Chelydra serpentina* (Common snapping turtle)	0.91732	0.9142	0.91211
*Crocodylus niloticus* (Nile crocodile)	0.91653	0.91448	0.91326
*Crotalus horridus* (Timber rattlesnake)	0.91366	0.91336	0.91345
*Naja naja* (Indian cobra)	0.91379	0.91192	0.913

**Table 3:** Results of the DFA exponent for all groups and each of the three random-walk rules.

**Scientific name (common name)**	**β_*RY*_**	**β_*SW*_**	**β_*KM*_**
*Ambystoma tigrinum tigrinum* (Eastern tiger salamander)	0.67836	0.90728	0.71664
*Bufo gargarizans* (Chusan Island toad)	0.75691	0.76766	0.76934
*Rana plancyi* (Eastern golden frog)	0.78803	0.74711	0.74653
*Ara ararauna* (Blue-and-yellow macaw)	0.74963	0.65734	0.86363
*Archilochus colubris* (Ruby-throated hummingbird)	0.74416	0.6971	0.86625
*Columba livia* (Rock pigeon)	0.76494	0.67966	0.86371
*Gallus gallus* (Red junglefowl)	0.7581	0.66958	0.87402
*Ninox strenua* (Powerful owl)	0.75282	0.6407	0.88804
*Carcharodon carcharias* (Great white shark)	0.74703	0.80776	0.79693
*Cyprinus carpio* (Common carp)	0.67192	0.75648	0.8331
*Dicentrarchus labrax* (European seabass)	0.75312	0.73671	0.72254
*Poecilia reticulata* (Guppy)	0.73809	0.78308	0.78935
*Didelphis virginiana* (Virginia Opossum)	0.70386	0.90263	0.7744
*Macropus giganteus* (Eastern gray kangaroo)	0.73178	0.8363	0.84188
*Vombatus ursinus* (Common wombat)	0.72691	0.83327	0.85255
*Bos taurus* (Cattle)	0.69678	0.84215	0.82662
*Canis lupus familiari*s (Dog)	0.71743	0.84081	0.79415
*Capra aegagrus* (Wild goat)	0.69553	0.84634	0.83395
*Felis catus* (Domestic cat)	0.70012	0.82755	0.82021
*Mus musculus musculus* (House mouse)	0.68457	0.87555	0.82526
*Oryctolagus cuniculus* (Common rabbit)	0.7394	0.82727	0.80349
*Rattus rattus* (House rat)	0.70334	0.85943	0.82893
*Gorilla gorilla gorilla* (Western lowland gorilla)	0.76455	0.7491	0.85718
*Homo sapiens* (Human)	0.76264	0.73476	0.8657
*Lemur catta* (Ring-tailed lemur)	0.72169	0.86066	0.81856
*Pan paniscus* (Bonobo)	0.76222	0.75973	0.86114
*Pan troglodytes* (Common chimpanzee)	0.76283	0.75342	0.86122
*Alligator mississippiensis* (American alligator)	0.74351	0.76308	0.84625
*Chelydra serpentina* (Common snapping turtle)	0.68238	0.85194	0.83671
*Crocodylus niloticus* (Nile crocodile)	0.69992	0.75112	0.83504
*Crotalus horridus* (Timber rattlesnake)	0.70735	0.75833	0.86203
*Naja naja* (Indian cobra)	0.69597	0.79567	0.85368

In Figure 2, there is an initial upward trend that is present irrespective of the species. The *RY* rule (Equation 2) implies that a (local) inclination toward the positive direction of the vertical axis corresponds to a (local) majority of purines (adenine or guanine). Similarly, the downward trend in Figure 3 reflects a consistent predominance of the weakly-pairing bases, adenine or thymine (considering rule *SW*). Thus, adenine dominates within the range 0− ~ 3, 000 bp.

The Hurst exponents for the rules *RY, SW*, and *KM* (Equations 2–4, respectively) fall in the range of 0.900−0.912 and imply a long-term positive autocorrelation. To put it into perspective, a Hurst exponent value of 0.9 indicates that, on average, the tendency of changes between nucleotides varies slightly as the sub-sequence size is changed. Moreover, the proximity of the Hurst exponent toward unity suggests that either purines or pyrimidines are predominant; it cannot distinguish, however, which one prevails. Similarly, the DFA exponents fall within 0.64−0.91 which implies the existence of strong long-range correlations in the sequences even after detrending. Interestingly, neither the Hurst nor DFA exponent values are near zero in any of the species considered. A possible explanation is that the tendency of changes between nucleotides does not vary randomly; i.e., mtDNA has an informational structure.

For all the DNA sequences, the Chargaff ratio is positive with ξ > 1, implying a larger amount of pyrimidines than purines. This implication is visually reflected in the overall downward tendency of the curves in Figure 2.

The disequilibrium coefficient takes values D∈(0.01-0.03). From Equation (12), values near 0 imply that the probabilities $p_{i}\approx \frac{1}{4}$ for any of the four nucleic bases. In other words, the disequilibrium values obtained suggest that the four nucleotide bases appear with almost the same proportion within each of the 32 mtDNA sequences. This is further supported by the Shannon entropy values. In this case, Equation (10) and *N* = 4 yield a (theoretical) maximum entropy value $H=\log_{2}\left(4\right)=2$. Hence, the empirical entropy values H∈(1.89-1.97) suggest near-equiprobability among the nucleic bases.

A graph of D vs. the Shannon entropy H suggests a linear relation. On this account, the disequilibrium coefficient is omitted for the remainder of the study. In addition, the complexity coefficient is omitted due to its direct proportionality to D. See Figure S4 (Supplementary Material).

This work proposes three new evolutionary indices as functions of Shannon entropy, the Chargaff ratio, and the fractal dimensions derived from the Hurst and DFA exponents:

(14) $$v_{1}\;=H*\log\left[\;\alpha^{\prime }{}_{RY}*\xi *\log\;\left(\alpha^{\prime }{}_{KM}\right)\right]$$

(15) $$\begin{array}{r} v_{2}=\log\left[\beta_{RY}^{{}^{\prime }}*\log\left(\beta_{KM}^{{}^{\prime }}\right)\right] \end{array}$$

(16) $$\begin{array}{r} v_{3}=\log\left[\beta_{SW}^{{}^{\prime }}*\log\left(\alpha_{SW}^{{}^{\prime }}\right)\right]. \end{array}$$

These indices reflect the long-range correlations found in DNA-walks and the information given by Shannon entropy and the Chargaff ratio.

The fractal dimensions α′ and β′ are derived from the Hurst and DFA exponents, respectively, using Equation (5). The natural logarithm can be seen as a transformation that maximizes the differences between the coefficients. Equations (14), (15), and (16) are defined from an evolutionary perspective, while Equation (16) provides information on the energy content of sequences.

In Equation (14), the logarithm of the fractal dimension derived from the Hurst exponent using the *KM* rule provides information regarding the transversions and transitions of the entire DNA sequence. On the other hand, the Chargaff ratio is used as a weighting factor for the fractal dimension derived using the *RY* rule. The logarithm of the product of these quantities provides an evolutionary measure related to the long-range correlations. The last term in the equation (the Shannon entropy) evaluates the probability of independent nucleotide changes for a given DNA sequence.

Equation (15) uses the fractal dimensions of the DFA exponents, which are computed using the detrended DNA-walks. Therefore, it is not accurate to include the Chargaff ratio or Shannon entropy as normalization parameters. Finally, Equation (16) represents a measure of the natural selection factors in relation to the environment. Results for *v*_1_, *v*_2_, *v*_3_ are shown in Table 4.

**Table 4:** New variables.

**Scientific name (common name)**	***v*_1_**	***v*_2_**	***v*_3_**
*Ambystoma tigrinum tigrinum* (Eastern tiger salamander)	−4.31380	−1.10950	−2.39820
*Bufo gargarizans* (Chusan Island toad)	−4.23240	−1.35480	−2.26260
*Rana plancyi* (Eastern golden frog)	−4.09750	−1.29530	−2.25390
*Ara ararauna* (Blue-and-yellow macaw)	−4.23570	−1.83360	−2.19300
*Archilochus colubris* (Ruby-throated hummingbird)	−4.27030	−1.84760	−2.21910
*Columba livia* (Rock pigeon)	−4.21960	−1.84640	−2.20990
*Gallus gallus* (Red junglefowl)	−4.17150	−1.91490	−2.17750
*Ninox strenua* (Powerful owl)	−4.21900	−2.02220	−2.21770
*Carcharodon carcharias* (Great white shark)	−4.18720	−1.46250	−2.31750
*Cyprinus carpio* (Common carp)	−4.47010	−1.58480	−2.28400
*Dicentrarchus labrax* (European seabass)	−4.35900	−1.18640	−2.12810
*Poecilia reticulata* (Guppy)	−4.20920	−1.42200	−2.30390
*Didelphis virginiana* (Virginia Opossum)	−4.29970	−1.33300	−2.40300
*Macropus giganteus* (Eastern gray kangaroo)	−4.28390	−1.68110	−2.34200
*Vombatus ursinus* (Common wombat)	−4.31840	−1.74230	−2.33970
*Bos taurus* (Cattle)	−4.37060	−1.56840	−2.34500
*Canis lupus familiaris* (Dog)	−4.28210	−1.42680	−2.35010
*Capra aegagrus* (Wild goat)	−4.35320	−1.60750	−2.34750
*Felis catus* (Domestic cat)	−4.39050	−1.53750	−2.34000
*Mus musculus musculus* (House mouse)	−4.33330	−1.55190	−2.37410
*Oryctolagus cuniculus* (Common rabbit)	−4.24440	−1.48650	−2.33690
*Rattus rattus* (House rat)	−4.33380	−1.58590	−2.35240
*Gorilla gorilla gorilla* (Western lowland gorilla)	−4.16280	−1.80220	−2.27510
*Homo sapiens* (Human)	−4.16480	−1.85860	−2.26910
*Lemur catta* (Ring-tailed lemur)	−4.24340	−1.54580	−2.36720
*Pan paniscus* (Bonobo)	−4.15450	−1.82670	−2.28690
*Pan troglodytes* (Common chimpanzee)	−4.15590	−1.82770	−2.28130
*Alligator mississippiensis* (American alligator)	−4.26190	−1.71650	−2.26170
*Chelydra serpentina* (Common snapping turtle)	−4.37120	−1.61300	−2.35910
*Crocodylus niloticus* (Nile crocodile)	−4.44740	−1.61700	−2.27800
*Crotalus horridus* (Timber rattlesnake)	−4.31660	−1.78940	−2.27140
*Naja naja* (Indian cobra)	−4.35360	−1.72560	−2.28610

**Figure 5:**
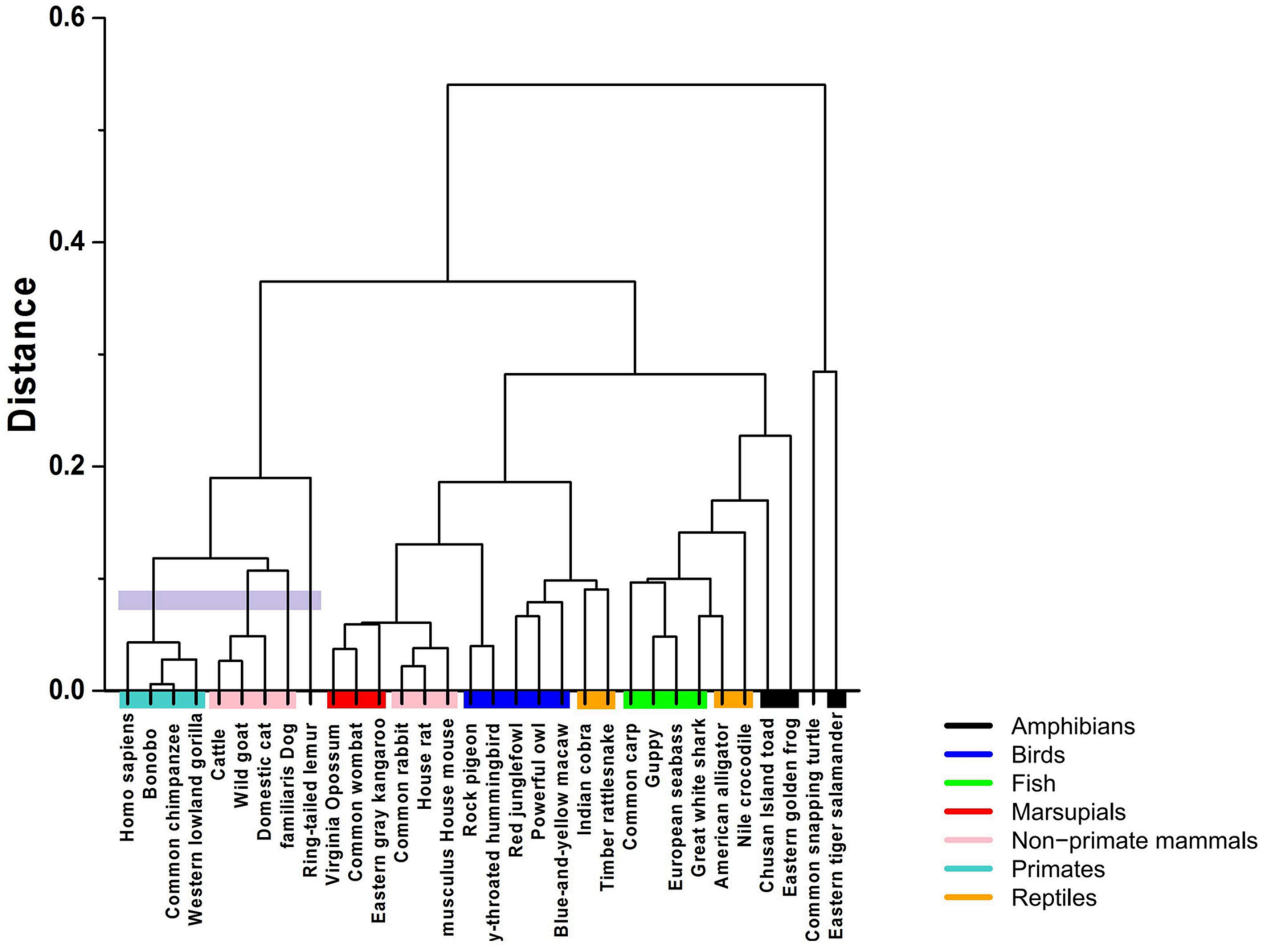
Hierarchical clustering of the 32 species using the Hurst exponent metric with and without tendency, weighted by the Chargaff ratio and Shannon entropy.

**Figure 6:**
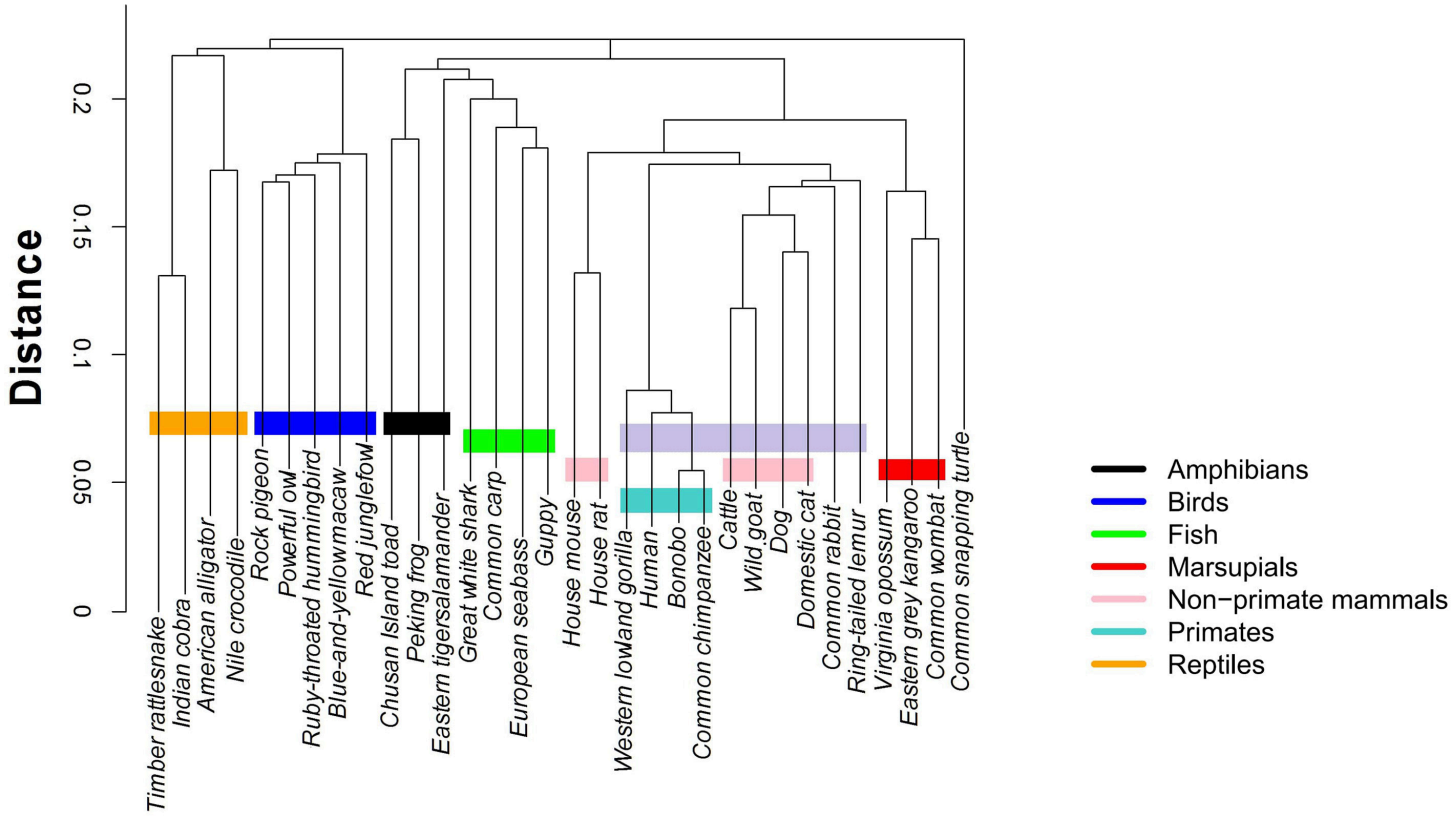
Hierarchical clustering of the 32 species using ClustalW https://www.ebi.ac.uk/Tools/msa/clustalo/.

Clustering algorithms may benefit from the proposal. Preliminary results, shown in Figure 5, suggest a possible application in studies centering on the evolutionary relations among species. The proposed indices are used in the *group-average agglomerative clustering* algorithm with Euclidean metric and the sum of distances as the clustroid. Furthermore, an additional grouping was constructed using a traditional program, ClustalW, which is frequently applied to the study of phylogenetic trees, as seen in Figure 6.

The implementation of the algorithm using the R programming language is not computationally demanding, with running times of about 15–20 min. In comparison, ClustalW requires about 2 and a half hours for the construction of the phylogenetic tree of 32 mtDNA sequences.

The comparative analysis between the two methods shows consistency among the group of primates and other mammals sharing a common ancestry of similar lineage to the lemur. On the other hand, the marsupials and rodents (including the common rabbit) are more closely grouped with the stochastic algorithm and present a common ancestor, just as calculated by the traditional method. Other groups that share proximity with both methods are the reptiles and the birds, as well as the fish group and some amphibians.

The most pronounced differences are found in certain taxa. The proposed method relates the rabbit more closely to rodents, with characteristics similar to marsupials. Meanwhile, the traditional method positions the rabbit closer to primates. Another interesting point is that the proposed stochastic method shows that small reptiles and birds are more closely related, while the traditional method relates the birds closer to large reptiles.

## 4. Conclusions

As has been suggested by other studies, Shannon entropy and Hurst and DFA exponents provide insight into the properties of DNA sequences (Peng et al., 1994; Oiwa and Glazier, 2004; Melnik and Usatenko, 2014; Monge and Crespo, 2015; Namazi and Kiminezhadmalaie, 2015; Salgado-Garcia and Ugalde, 2016; Thanos et al., 2018). This exploratory analysis combines various measures utilized in the literature to establish a biologically meaningful measure of distinction among species.

Our proposal defines new indices as functions of Shannon entropy, the Chargaff ratio, and fractal dimensions using rescaled-range analysis and DFA. These indices can be employed to construct phylogenetic trees using clustering algorithms.

Long-range correlations attributed to DNA-walks can be identified during our study. These can represent data with persistence in its evolutionary memory; i.e., that mtDNA sequences contain highly conserved regions among similar species.

The comparison between the traditional and the proposed clustering method shows clear agreements; however, there are differences that must be analyzed under an evolutionary perspective. For example, we notice that the mtDNA sequences of the common rabbit and the common snapping turtle show different properties in both methods. According to the established phylogeny, the placement of the rabbit is closer to the rodents. Interestingly, results of the stochastic hierarchical clustering suggest a potential application for phylogenetic studies.

Evolutionary processes are associated to an adaptive selection of the species throughout millions of years. However, the fluctuations of the changes in nucleotide bases could be random in order to find new sequence combinations. The proposed method attempts to measure the stochastic fluctuations to yield indices that allow the observation of tendencies and correlations in the mutations that produce new species throughout evolutionary history.

## Author Contributions

MC-R provided data collection of the mtDNA sequences from the GenBank^®^, worked on numerical and graphical results, and drafted the article. FJ provided numerical analysis, methodology, and mathematical insight. FH-C rendered numerical analysis, as well as mathematical and biological interpretations. JC-G contributed with numerical analysis, revision, critical revision for important intellectual content, and co-final approval of the version to be published. OG-A provided textual and structural revision of the co-final version of this work.

### Conflict of Interest Statement

The authors declare that the research was conducted in the absence of any commercial or financial relationships that could be construed as a potential conflict of interest.
