# Supplementary material for: A Stochastic Phylogenetic Algorithm for Mitochondrial DNA Analysis
Source: Front Genet. 2019 Mar 8;10:66. doi: 10.3389/fgene.2019.00066 (PMC6418022; doi:10.3389/fgene.2019.00066)
Supplement: Supplementary file 1 [file Data_Sheet_1.pdf]

Supplementary Material:
A stochastic phylogenetic algorithm for mitochondrial DNA analysis

SUPPLEMENTARY TABLES AND FIGURES

1 Tables

Table S1: NCBI codes for the 32 species considered in this study.

Scientific name (Common name)	NCBI Code
Ambystoma tigrinum tigrinum (Eastern tiger salamander)	NC_006887.1
Bufo gargarizans (Chusan Island toad)	NC_008410.1
Rana plancyi (Eastern golden frog)	NC_009264.1
Ara ararauna (Blue-and-yellow macaw)	NC_029319.1
Archilochus colubris (Ruby-throated hummingbird)	NC_010094.1
Columba livia (Rock pigeon)	NC_013978.1
Gallus gallus (Red junglefowl)	NC_001323.1
Ninox strenua (Powerful owl)	NC_033967.1
Carcharodon carcharias (Great white shark)	NC_022415.1
Cyprinus carpio (Common carp)	NC_001606.1
Dicentrarchus labrax (European seabass)	NC_026074.1
Poecilia reticulata (Guppy)	NC_024238.1
Didelphis virginiana (Virginia Opossum)	NC_001610.1
Macropus giganteus (Eastern gray kangaroo)	NC_027424.1
Vombatus ursinus (Common wombat)	NC_003322.1
Bos taurus (Cattle)	NC_006853.1
Canis lupus familiaris (Dog)	NC_002008.4
Capra aegagrus (Wild goat)	NC_028161.1
Felis catus (Domestic cat)	NC_001700.1
Mus musculus musculus (House mouse)	NC_010339.1
Oryctolagus cuniculus (Common rabbit)	NC_001913.1
Rattus rattus (House rat)	NC_012374.1
Gorilla gorilla gorilla (Western lowland gorilla)	NC_011120.1
Homo sapiens (Human)	NC_012920.1
Lemur catta (Ring-tailed lemur)	NC_004025.1
Pan paniscus (Bonobo)	NC_001644.1
Pan troglodytes (Common chimpanzee)	NC_001643.1
Alligator mississippiensis (American alligator)	NC_001922.1
Chelydra serpentina (Common snapping turtle)	NC_011198.1
Crocodylus niloticus (Nile crocodile)	NC_008142.1
Crotalus horridus (Timber rattlesnake)	NC_014400.1
Naja naja (Indian cobra)	NC_010225.1

2 Figures

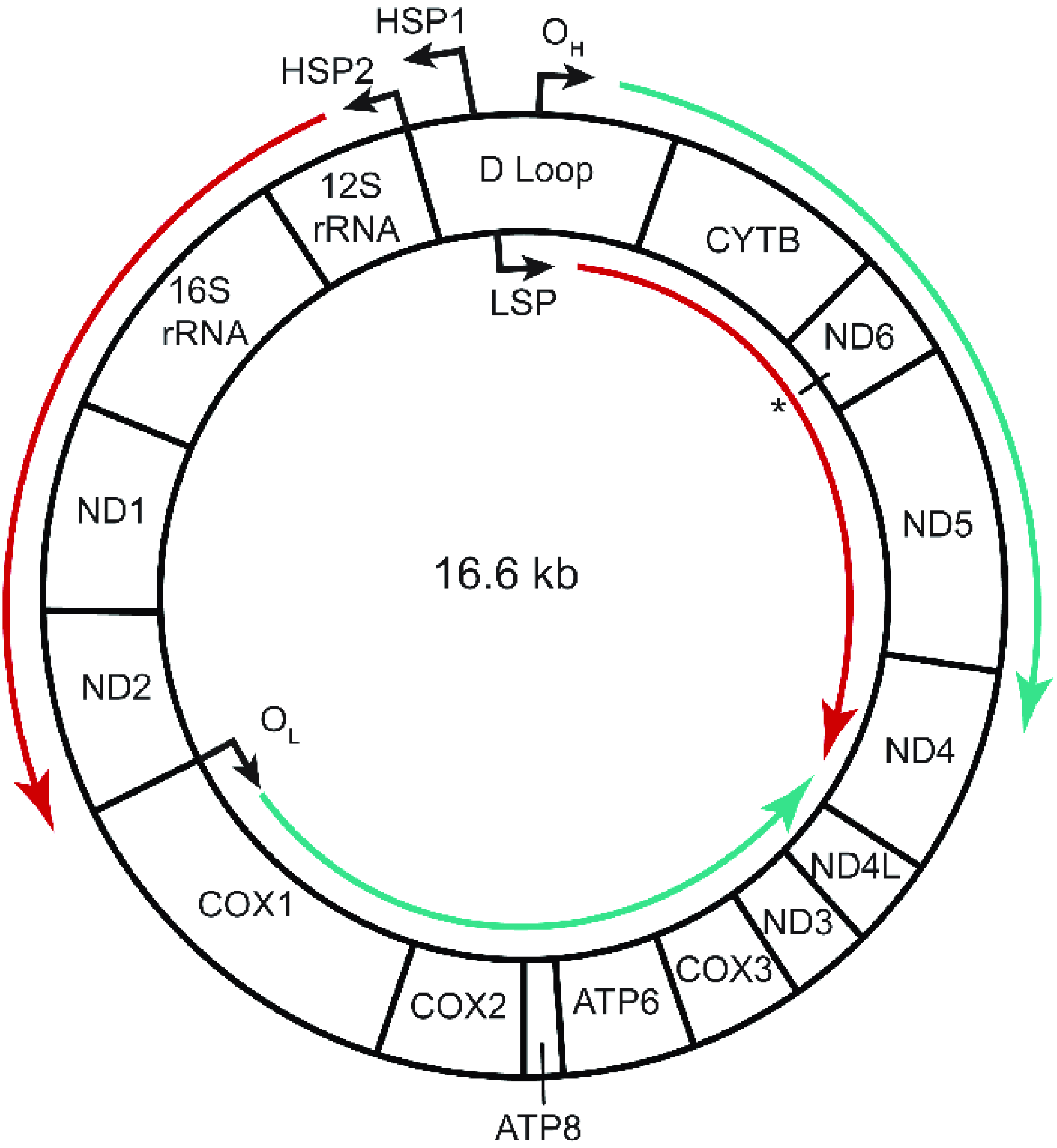

Figure S1: Mitochondrial DNA depletion by ethidium bromide decreases neuronal mitochondrial creatine kinase: Implications for striatal energy metabolism - Scientific Figure on ResearchGate. Available from: https://www.researchgate.net/The-mitochondrial-genome-mtDNA-rRNA-encoding-and-protein-encoding-genes-are-shown_fig1_322143416 [accessed 23 Jul, 2018]

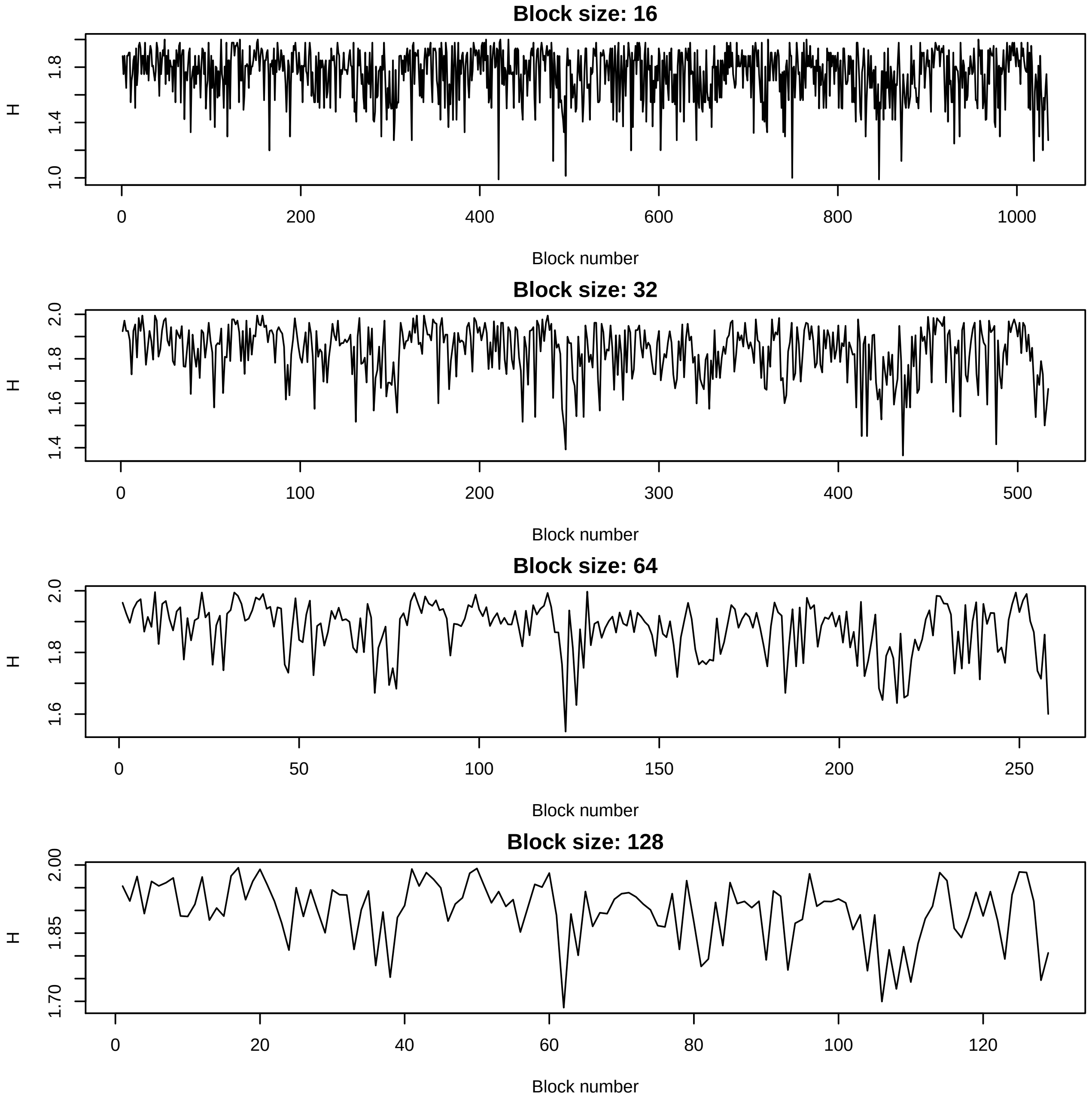

Figure S2: Shannon Entropy fluctuations at different window sizes. The minimum entropy value increases in direct relation with window size. Interestingly, the pattern and overall shape remains remarkably similar across scales. This prompts the use of fractal analysis, here left for future research.

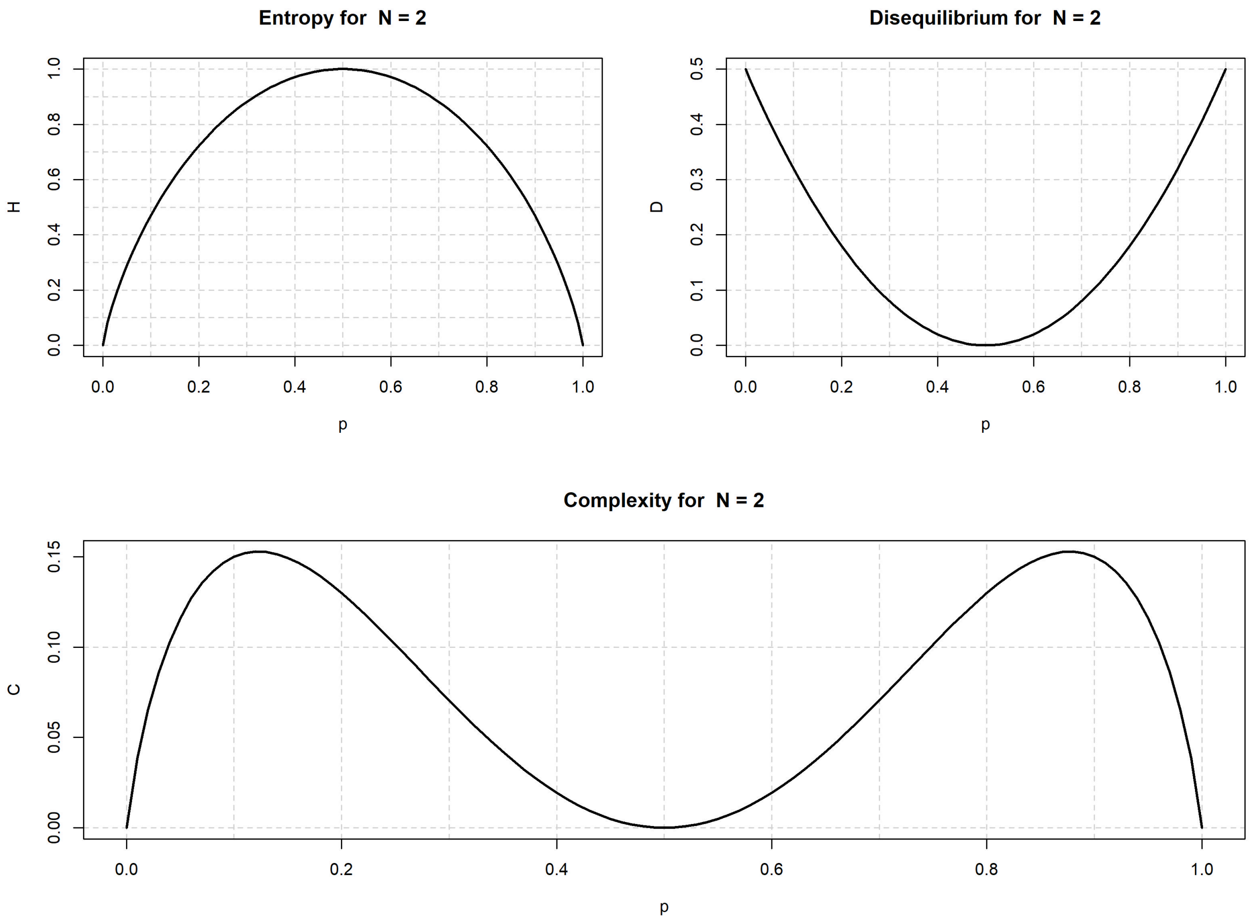

Figure S3: Shannon entropy, disequilibrium, and complexity values for a system of $N = 2$ events with occurrence probabilities p and $1 - p$.

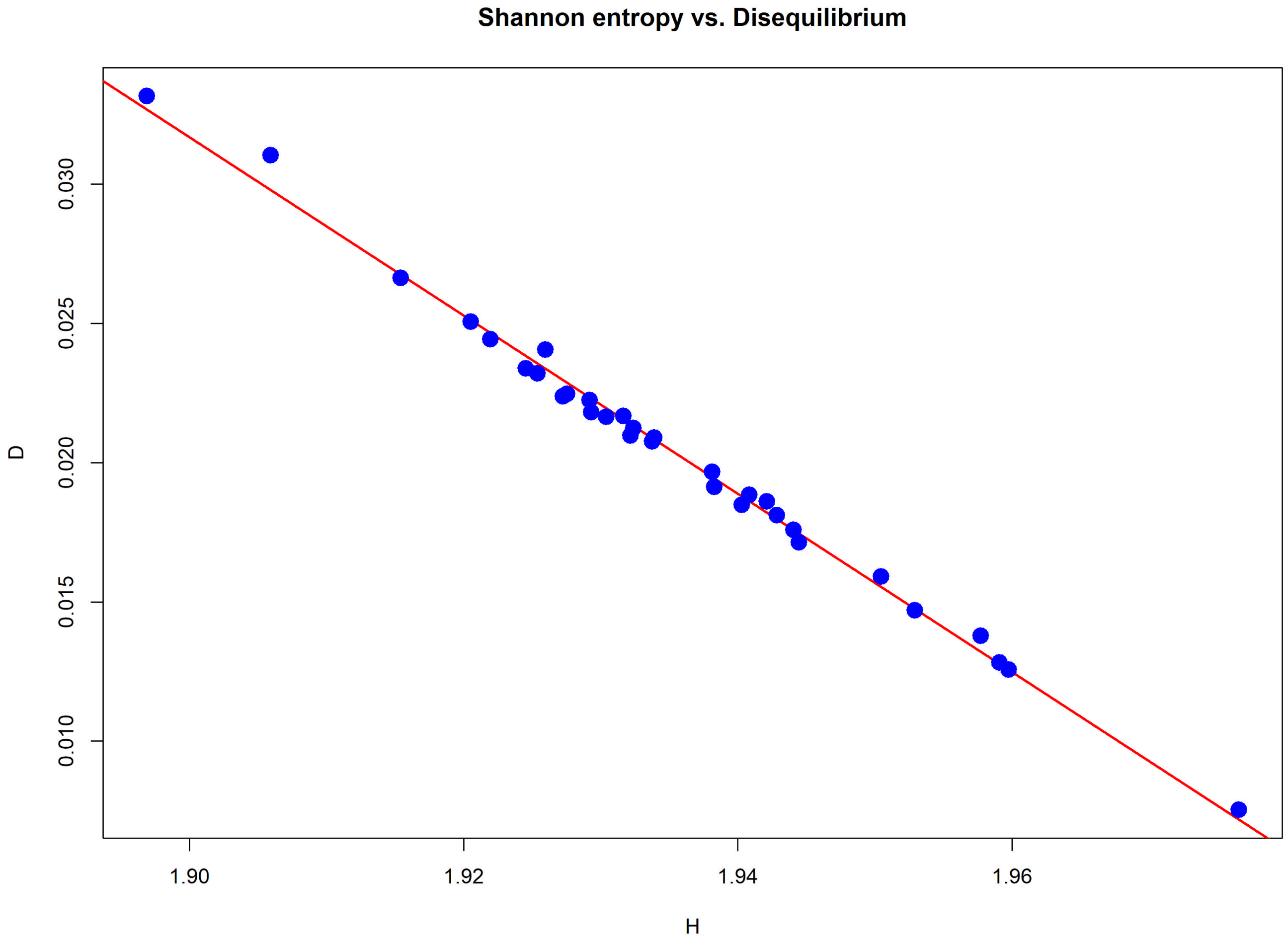

Figure S4: Blue dots depict the relation between Shannon entropy and disequilibrium coefficients. The red line is a linear fit with slope value of $-.32$.
